# Recombinant Mutated Human TNF in Combination with Chemotherapy for Stage IIIB/IV Non-Small Cell Lung Cancer: A Randomized, Phase III Study

**DOI:** 10.1038/srep09918

**Published:** 2015-04-21

**Authors:** Xiaowen Ma, Yang Song, Kuo Zhang, Lei Shang, Yuan Gao, Wei Zhang, Xiaochang Xue, Huimin Jia, Jian Geng, Wei Zhou, Yazheng Dang, Enxiao Li, Xinyu Ti, Fulin Fan, Yingqi Zhang, Meng Li

**Affiliations:** 1 State Key Laboratory of Cancer Biology, Biotechnology Center, School of Pharmacy, The Fourth Military Medical University, Xi'an, China; 2 Department of Oncology, Tangdu Hospital, The Fourth Military Medical University, Xi'an, China; 3 Department of Health Statistics, School of Public Health, The Fourth Military Medical University, Xi'an, China; 4 The Affiliated Cancer Hospital of Xinjiang Medical University, Urumqi, China; 5 Department of Medical Oncology, General Hospital of Nanjing Military Command, Medical School of Nanjing University, Nanjing, China; 6 General Hospital of Ningxia Medical University, Yinchuan, China; 7 Cancer Center, The 323 Hospital of People's Liberation Army, Xi'an, China; 8 Department of Medical Oncology, First Affiliated Hospital of Medical College, Xi'an Jiaotong University, Xi'an, China; 9 Department of Respiratory Medicine, Xijing Hospital, The Fourth Military Medical University, Xi'an, China; 10 New Taihe Biopharmaceutical Co., Ltd., Guangzhou, China; 11 Department of Pharmacogenomics, School of Pharmacy, The Fourth Military Medical University, Xi'an, China

## Abstract

Tumor necrosis factor (TNF), an anti-angiogenic agent in cancer treatment, is limited to isolated limb perfusion due to systemic toxicities. We previously prepared a TNF mutant (rmhTNF) that significantly improved responses in lung cancer patients and exhibited a promising safety profile in phase I and II studies. To further investigate whether rmhTNF with standard chemotherapy provides a survival benefit, 529 patients with stage IIIB/IV non-small cell lung cancer (NSCLC) were randomly assigned to receive docetaxel plus carboplatin/cisplatin with rmhTNF (265) or chemotherapy alone (264). After four cycles of treatment, the median overall survival was 13.7 months in the chemotherapy plus rmhTNF group compared with 10.3 months in the chemotherapy group (hazard ratio (HR) 0.75, *P* = 0.001). The median progression-free survival in the chemotherapy plus rmhTNF group and the chemotherapy group was 8.6 and 4.5 months (HR 0.76, *P* = 0.001), respectively, with corresponding response rates of 38.5% and 27.7% (*P* = 0.008). Increased hyperpyrexia and pulmonary hemorrhage were associated with rmhTNF, but most effects were well tolerated. The results indicated that rmhTNF effectively potentiated chemotherapy in patients with advanced NSCLC and was comparable with bevacizumab, an angiogenesis inhibitor approved by the Food and Drug Administration (FDA) for NSCLC.

Non-small cell lung cancer (NSCLC), a leading cause of cancer death, is often diagnosed at advanced stages when few treatment options are available^1^,^2^. Although modest progress has been made with the use of platinum-based combination chemotherapy, additional treatment methods are needed^3^,^4^. Angiogenesis is a hallmark of cancer^5^. Anti-angiogenic therapy can destroy or ‘normalize' excessive and abnormal blood vessels in the tumor, thereby potentiating the effects of chemotherapy by improving the delivery of drugs and oxygen^6^. Bevacizumab (Avastin, Genentech/Roche, San Francisco, CA, US), a monoclonal antibody against vascular endothelial growth factor (VEGF), was approved by the Food and Drug Administration (FDA) for the treatment of stage IIIB/IV non-squamous NSCLC in combination with paclitaxel and carboplatin in 2006^7^. The approval and the associated pivotal study proved that anti-angiogenic therapy is useful in advanced NSCLC^8^,^9^.

Tumor necrosis factor (TNF) is a monocyte-derived cytokine that stimulates the acute phase reaction of the immune system. Its abilities to destroy tumor vasculature, induce hemorrhagic necrosis in specific tumor types and synergize with various chemotherapy reagents were established two decades ago^10^,^11^,^12^. However, as an immune stimulator and endogenous pyrogen, TNF is implicated in septic shock, cachexia and fever^13^,^14^. The maximum tolerable dose of TNF in patients is 10-fold lower than the effective antitumor dose^15^,^16^,^17^. The clinical application of TNF has been limited to the isolated limb perfusion (ILP) setting for soft tissue sarcoma (STS) and melanoma in-transit metastases confined to the limb. Systemic toxicity is abolished by limb isolation and the resulting extra-corporeal circulation^18^,^19^. An approximately 100% response rate acquired by ILP with TNF and melphalan in patients with STS has inspired various strategies to minimize the toxicities of TNF for its application as a systemic anti-cancer drug^20^,^21^,^22^.

We previously prepared a recombinant mutated human TNF (rmhTNF, NAX®) featuring the deletion of the first seven amino acids and substitution of four amino acids (Arg for Pro at position 8, Lys for Ser at position 9, Arg for Asp at position 10, and Phe for Leu at position 157). rmhTNF exhibits 25-fold increased antitumor effects and at least a 50-fold increased LD50 (50% lethal dose) compared with wild type TNF^23^,^24^. In previous phase I and II studies, rmhTNF plus chemotherapy achieved a 48.89% response rate compared with 17.78% for chemotherapy alone (*P* < 0.001) in patients with advanced NSCLC, and most adverse events (AEs) were well tolerated^25^.

The NCD-ANSCLC (NAX® with Carboplatin/Cisplatin and Docetaxel in Advanced Non-Small Cell Lung Cancer) phase III trial was conducted to confirm phase II results. The primary objective was to compare overall survival (OS) of the rmhTNF and chemotherapy combination with standard chemotherapy. Progression-free survival (PFS), response rate (RR), survival rates and safety assessments were also reported.

## Results

An independent data monitoring committee reviewed the data and recommended the release of the study results in March 2012, given that the criteria for significance pre-specified in the protocol had been achieved. The results reported here include follow-up through December 2012 (median follow-up was 15.8 months for the chemotherapy plus rmhTNF group and 13.6 months for the chemotherapy alone group). The patients lost during follow-up were included in disease progression or death data.

### Population Characteristics

**Figure 1:**
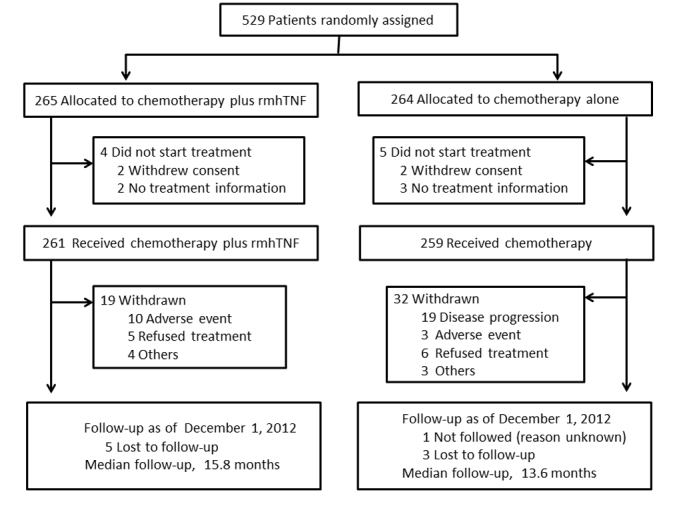
Enrollment, randomization, and follow-up of study patients. Chemotherapy = docetaxel plus carboplatin or cisplatin; rmhTNF, recombinant mutated human tumor necrosis factor.

Between January 2007 and April 2010, 529 patients were randomly assigned to receive chemotherapy plus rmhTNF (n = 265) or chemotherapy alone (n = 264). Demographic and baseline characteristics were balanced between the treatment arms (Table 1). No significant differences were noted regarding patient characteristics in various strata. In addition, 19 and 32 patients discontinued treatment in the chemotherapy plus rmhTNF and chemotherapy groups, respectively, due to disease progression, adverse events, refusal or other reasons (Fig. 1). Five patients in the chemotherapy plus rmhTNF group and 3 patients in the chemotherapy group were lost to follow-up. Only 1 patient in the chemotherapy group was not followed. The most common follow-up therapy administered was chemotherapy (post-study treatment). More patients in the chemotherapy group (70%) received follow-up therapies than in the chemotherapy plus rmhTNF group (58%).

### Efficacy

**Figure 2:**
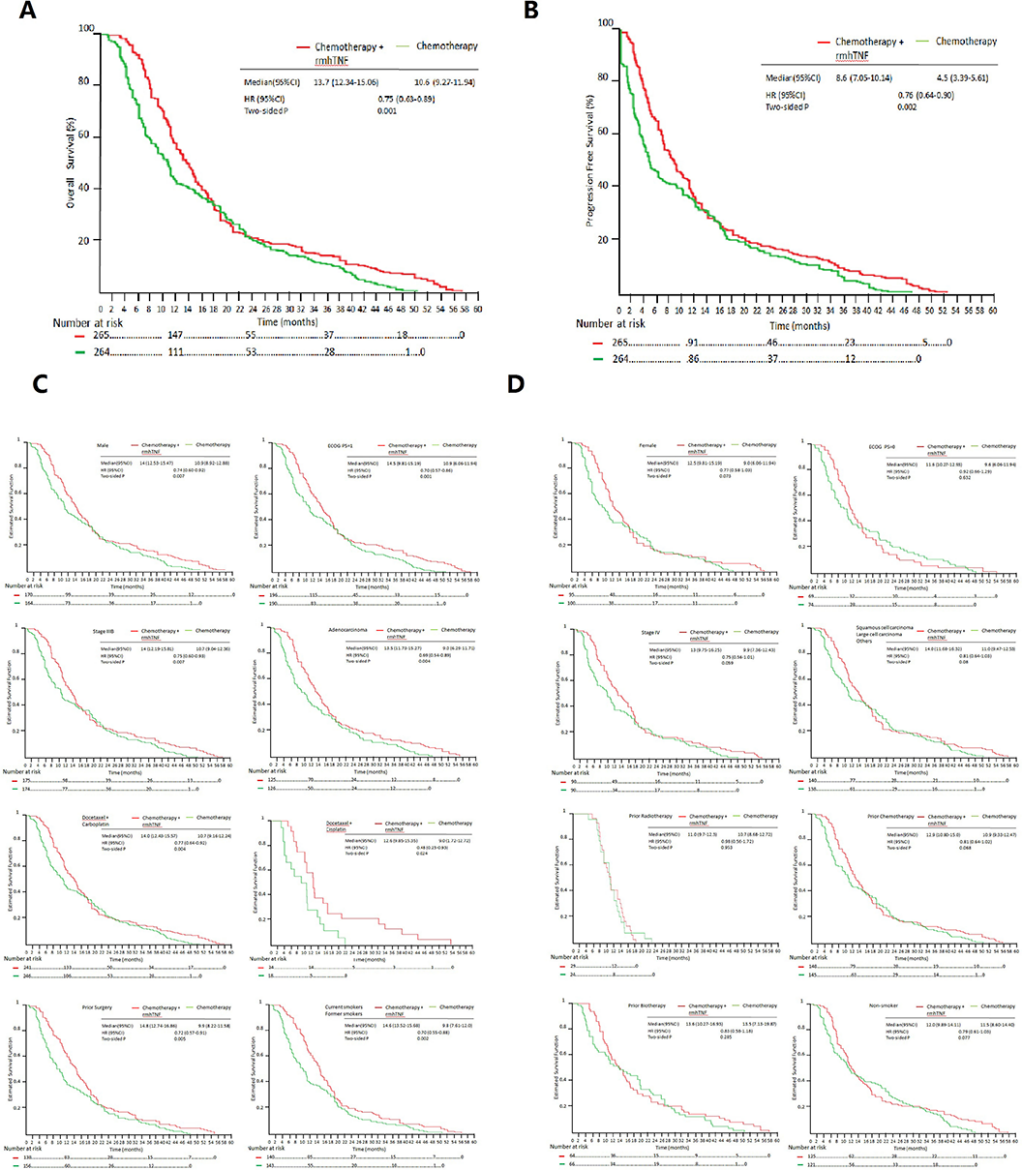
Kaplan-Meier curves of the pre-specified stratification populations. (A) Overall survival in the intention-to-treat population. (B) Progression-free survival in the intention-to-treat population. (C) Overall survival in populations whose median survival exhibited significant differences after rmhTNF plus chemotherapy treatment or chemotherapy alone (*P* < 0.05). (D) Overall survival in the populations whose median survival did not exhibit significant differences after rmhTNF plus chemotherapy treatment or chemotherapy alone (*P* > 0.05). Chemotherapy = docetaxel plus carboplatin or cisplatin; rmhTNF, recombinant mutated human tumor necrosis factor; CI, confidence intervals.

The primary analysis consisted of all randomly assigned patients (the intention-to-treat population, ITT). The median OS was 13.7 months [95% confidence intervals (CI) 12.34–15.06] for the chemotherapy plus rmhTNF group compared with 10.6 months [95% CI 9.27–11.94] for the chemotherapy group (hazard ratio, HR 0.75 [95% CI 0.63–0.89], *P* = 0.001, Fig. 2A). The median PFS was 8.6 months [95% CI 7.05–10.14] for the chemotherapy plus rmhTNF group and 4.5 months [95% CI 3.39–5.61] for the chemotherapy group (HR 0.76 [95% CI 0.64–0.90], *P* = 0.001, Fig. 2B). Secondary end points including RR, response duration and survival rates are presented in Table 2. In total, 102 (38.5%) patients in the chemotherapy plus rmhTNF group and 73 (27.7%) in the chemotherapy group exhibited a CR (complete response) or PR (partial response). The RR of the combination group was significantly increased compared with the chemotherapy group (odd ratio (OR) 1.64 [95% CI 1.14–2.36], *P* = 0.008). The median response duration was 5.8 months (interquartile range (IQR) 3.6–11) for the chemotherapy plus rmhTNF group and 4.1 months (IQR 2.8–9.8) for the chemotherapy group. At 1 year, 147 (55.5%) patients in the chemotherapy plus rmhTNF group survived compared with 111 (42%) in the chemotherapy group (OR 1.72 [95% CI 1.22–2.42], *P* = 0.002). At 4 years, 18 (6.8%) patients in the chemotherapy plus rmhTNF group survived compared with 1 (0.4%) in the chemotherapy group (OR 19.17 [95% CI 2.54–144.65], *P* < 0.001). No significant differences were observed between the survival rates of the two arms at 2 and 3 years.

### Subgroup Analyses

**Figure 3:**
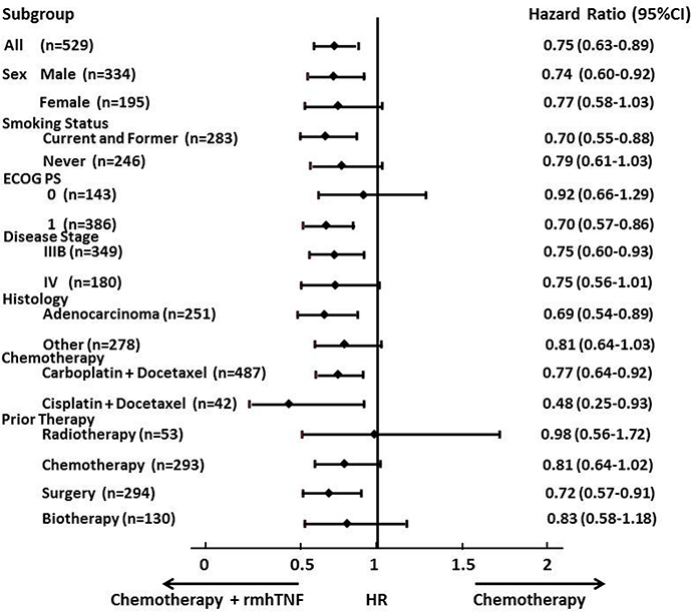
Forest plot of hazard ratios for overall survival assessed by subgroup factors. Horizontal lines represent confidence intervals (CI). ECOG PS, Eastern Cooperative Oncology Group Performance Status.

Regarding male patients, the median OS of the chemotherapy plus rmhTNF group was 14.0 months [95% CI 12.53–15.47] compared with 10.9 months [95% CI 8.92–12.88] in the chemotherapy group (HR 0.74 [95% CI 0.60–0.92], *P* = 0.007). For current and former smokers (who stopped smoking less than 5 years ago), the median OS of the chemotherapy plus rmhTNF group was 14.6 months [95% CI 13.52–15.68] compared with 9.8 months [95% CI 7.61–12.0] in the chemotherapy group (HR 0.70 [95% CI 0.55–0.88], *P* = 0.002). In PS 1 (European Cooperative Oncology Group, ECOG performance status) patients, the median OS of the chemotherapy plus rmhTNF group was 14.5 months [95% CI 9.81–15.19] compared with 10.9 months [95% CI 6.06–11.94] in the chemotherapy group (HR 0.70 [95% CI 0.57–0.86], *P* = 0.001). For patients with stage IIIB disease, the median OS of the chemotherapy plus rmhTNF group was 14.0 months [95% CI 12.19–15.81] compared with 10.7 months [95% CI 9.04–12.36] in the chemotherapy group (HR 0.75 [95% CI 0.60–0.93], *P* = 0.007). In patients with adenocarcinoma, the median OS of the chemotherapy plus rmhTNF group was 13.5 months [95% CI 11.73–15.27] compared with 9.0 months [95% CI 6.29–11.71] in the chemotherapy group (HR 0.69 [95% CI 0.54–0.89], *P* = 0.004). For patients who previously underwent surgery, the median OS of the chemotherapy plus rmhTNF group was 14.8 months [95% CI 12.74–16.86] compared with 9.9 months [95% CI 8.22–11.58] in the chemotherapy group (HR 0.72 [95%CI 0.57–0.91], *P* = 0.005). When carboplatin was administered, the median OS of the chemotherapy plus rmhTNF group was 14.0 months [95% CI 12.43–15.57] compared with 10.7 months [95% CI 9.16–12.24] in the chemotherapy group (HR 0.77 [95% CI 0.64–0.92], *P* = 0.004). When cisplatin was administered, the median OS of the chemotherapy plus rmhTNF group was 12.6 months [95% CI 9.85–15.50] compared with 9.0 months [95% CI 1.72-12.72] in the chemotherapy group (HR 0.48 [95% CI 0.25–0.93], *P* = 0.024). No significant differences were observed between the two arms for females, never smokers (including those who stopped smoking more than 5 years previously), PS 0 patients, patients with stage IV disease, and patients with diseases other than adenocarcinoma. In patients who previously received radiotherapy, chemotherapy and/or biological therapy, no significant differences were observed (Fig. 2C, 2D). However, HR analyses revealed that rmhTNF was beneficial in all the subgroups assessed if CIs were not considered. The HRs of the median OS based on patient stratification are summarized in Figure 3. The most significant benefit was noted in current and former smokers, patients with adenocarcinoma and patients administered cisplatin.

### Safety

All patients who received the study treatment (261 in the chemotherapy plus rmhTNF group and 259 in the chemotherapy group) were included in the analysis of toxic effects. Table 3 presents the rates of adverse events (AEs) in each treatment group. Most AEs were grade 1 or 2. Incidences of grade 3 and 4 pyrexia (drug-related fever), flu-like symptoms and hematemesis were significantly increased in chemotherapy plus rmhTNF group compared with the chemotherapy group (*P* < 0.05). These events were considered rmhTNF related. In the chemotherapy plus rmhTNF group, 7 patients withdrew due to pyrexia, and 3 withdrew due to neutropenia. In the chemotherapy group, 3 patients withdrew due to neutropenia. Diarrhea, rash, arrhythmia and albuminuria were the most common AEs noted in the two groups.

Fatal AEs were reported in ten patients. Seven were reported with chemotherapy plus rmhTNF, and three were reported with chemotherapy alone. Of the seven fatal AEs in the combination group, 3 were attributed to pulmonary hemorrhage (hematemesis), 2 to hypotension, 1 to pulmonary embolism and 1 to myocardial infarction. The three fatal AEs in the chemotherapy group included 2 cardiac events and 1 pulmonary embolism. Pulmonary hemorrhage and hypotension were considered rmhTNF related. Cardiac events were considered paclitaxel related. Other fatal AEs were deemed unrelated to the treatment.

## Discussion

Unlike normal blood vessels, tumor-associated vasculature is poorly organized and hyper-permeable^5^. As a result, interstitial hypertension exists within solid tumors. Interstitial hypertension and compression from cancer cells greatly compromises the delivery and effectiveness of conventional cytotoxic therapies^26^. Based on the death domain of the TNF receptor 1, TNF induces excessive apoptosis in endothelial cells and pericytes, which results in pruning of tumor vessels and a decrease in interstitial hypertension^27^. These effects facilitate the augmented distribution of the drug in the tumor and better exposure of the tumor cells to the cytostatic agent^18^,^28^. The addition of TNF-α improves the accumulation of chemotherapeutic drugs selectively in the tumor up to three- to six-fold in rat models. However, the wide expression of TNF receptors in the immune system and the downstream NF-κB stimulation signals caused uncontrollable hyperpyrexia when TNF-α was used as an anti-angiogenic agent in cancer treatment^29^. rmhTNF has a specific activity of 1 × 10^9^ unit/mg defined by the standard lysis method (actinomycin D-treated mouse L929 cells), which is at least 50-fold higher than TNF. The dose adopted in our studies (4 × 10^6^ unit/m^2^/day) was actually 4 µg/m^2^/day after undergoing calculations based on activity. Moderate side effects of rmhTNF were promised, as 100 to 400 µg/m^2^/day was considered effective for wild-type TNF^30^,^31^. In the present study, most side effects were well tolerated, and only 3 patients discontinued treatment due to fever. Pulmonary hemorrhage was another AE of special concern in our study. Hemorrhage is common in anti-angiogenic treatments. The incidence of life-threatening pulmonary hemorrhage was approximately 2% for bevacizumab in the treatment of patients with advanced, non-squamous NSCLC^8^. Therefore, hemoptysis was an exclusion criterion in the present study and should be monitored carefully.

Our results indicated that the addition of rmhTNF to a standard platinum-based chemotherapy regimen improved OS (13.7 months vs. 10.6 months, HR 0.75, *P* = 0.001). In addition, rmhTNF prolonged PFS and improved the RR and one-year survival rate. The benefits of rmhTNF were comparable to bevacizumab, which significantly improved the survival of patients with advanced NSCLC when combined with chemotherapy (12.3 months vs. 10.3 months, HR 0.79, *P* = 0.003)^8^. Patients in our study received four cycles of therapy, whereas most patients in bevacizumab trials were administered at least five cycles of combination treatment and long-term maintenance with monoclonal antibody. From the perspective of treatment costs, rmhTNF was more acceptable than monoclonal antibody for Chinese patients.

In the present study, OS improvements with rmhTNF were not consistent among all pre-specified stratification groups. Although the median OS was improved with rmhTNF plus chemotherapy versus chemotherapy alone, the differences were not statistically significant in some strata (Fig. 2D). However, HR analyses according to baseline characteristics indicated that rmhTNF was beneficial in all the subgroups assessed if CIs were not considered (Fig. 3). Possible explanations for this flaw include imbalances between the two groups with respect to known or unknown baseline prognostic factors, imbalances in the use of off-study therapies, statistical chance, or a true difference.

In conclusion, the addition of rmhTNF to a platinum-based, two-agent chemotherapy regimen conferred significant improvement in OS, PFS, RR and survival rate in NSCLC patients with a good performance status. Increased toxic effects, particularly hyperpyrexia and pulmonary hemorrhage, were associated with the addition of rmhTNF. These risks must be considered and monitored carefully when administering rmhTNF plus docetaxel and carboplatin/cisplatin for the treatment of patients with NSCLC.

## Patients and Methods

### Study Design and Population

NCD-ANSCLC was a multi-center, randomized, double-blind, phase III study comparing rmhTNF with docetaxel plus carboplatin or cisplatin versus chemotherapy alone in patients with stage IIIB/IV NSCLC. The study was performed in 6 hospitals in China. Eligible patients (≥ 18 years) were required to have histologically or cytologically confirmed stage IIIB/IV NSCLC. Other inclusion criteria were measurable lesions as defined by the Response Evaluation Criteria in Solid Tumors (RECIST version 3.0), a European Cooperative Oncology Group (ECOG) PS of 0 or 1, predicted life expectancy ≥ 12 weeks from first study-drug dose, and adequate hematologic, hepatic, cardiac and renal function. A washout period from previous treatments was required (2 to 6 weeks). Exclusion criteria included hemoptysis (1/2 tsp or more per event); central nervous system metastases; pregnancy or lactation; a history of documented hemorrhagic diathesis or coagulopathy; therapeutic anticoagulation; regular use of aspirin (> 325 mg/day), non-steroidal anti-inflammatory agents, or other agents known to inhibit platelet function; a history of hypersensitivity to paclitaxel, polypeptide drugs or biologics; and previous exposure to taxanes.

The study was approved by the ethics committee of each participating center (the Ethics Committee of West China Center of Medical Sciences, Sichuan University; the Ethics Committee of the Fourth Military Medical University; the Ethics Committee of Xinjiang Medical University; the Ethics Committee of Ningxia Medical University; the Ethics Committee of the First Affiliated Hospital of Medical College, Xi'an Jiaotong University; the Ethics Committee of the General Hospital of Nanjing Military Command) and complied with the Declaration of Helsinki and Good Clinical Practice guidelines. All patients provided written informed consent before any study-related procedure.

This trial is registered with Chictr.org (Chinese Clinical Trial Registry; number ChiCTR-IPR-14005500).

### Randomization and Masking

Research coordinators at each center randomly assigned eligible patients in a 1:1 ratio by use of a clinical trial randomization system^32^. The system could minimized imbalance between treatment groups within each stratum and generated a unique number for each patient. These numbers were reported to the hospital staff when patients were assigned. In turn, the hospital staff referred to a manual of unique numbers generated by an independent statistician prior to study activation to determine the study treatment allocated to the randomized patient. Study investigators, research coordinators, attending care teams, the patients and their families were blinded to treatment allocation.

### Procedures

Randomly assigned patients received four cycles of docetaxel (75 mg/m^2^ on day 1 of a 3 week cycle; intravenous (IV)) and platinum chemotherapy (carboplatin 5 × area under the curve or cisplatin 75 mg/m^2^ on day 1 of a 3 week cycle, IV) plus rmhTNF (4 × 10^6^ units?m^2^?day on days 1-7 and days 11-17 of each cycle, intramuscular (IM)) (chemotherapy plus rmhTNF group) or docetaxel and platinum (chemotherapy group). Treatment discontinuation or interruption due to AEs could occur at any time. Dosing could be interrupted for a maximum of 2 weeks if clinically indicated. At disease progression, treatment was unmasked. Patients in the chemotherapy group could continue or receive further treatment at their investigator's discretion. Patients in the chemotherapy plus rmhTNF group were required to discontinue treatment.

### Assessments and End Points

All patients were hospitalized for frequent monitoring of clinical signs during treatments. AEs and clinically significant laboratory abnormalities were recorded according to the National Cancer Institute Common Terminology Criteria for Adverse Events (version 3.0). After baseline evaluation, tumor responses were assessed by use of CT (computed tomography) with RECIST every 6 weeks for the treatment period, every 12 weeks for the first two years of follow-up, and then every 24 weeks until disease progression or death. An independent review committee of clinicians and radiologists masked to patient assignment reviewed all images and determined tumor response and progression status.

The primary efficacy end point was OS (the time from random assignment to death from any cause). Secondary end points included PFS (the time from randomization to documented disease progression or death), RR (CR+PR), response duration and survival rate.

### Statistical Analysis

All efficacy analyses were based on a comparison of the assigned treatments. The primary analysis comprised all randomly assigned patients (ITT). The safety population consisted of all patients who received at least one dose of the study drug and were subject to at least one post-baseline safety assessment. Based on an OS of 10.3 months in the control group of the previous study^8^, approximately 382 deaths were required to detect a HR of 0.75 (chemotherapy plus rmhTNF vs. chemotherapy alone) at 80% power with a two-sided log-rank test and an α-level of 5%. Accounting for patient ineligibility and withdrawal (approximately 15%), 529 patients were required.

Event-time distributions were estimated by the Kaplan-Meier method. Cox proportional-hazards models, stratified according to the disease stages, tumor histology, smoking status, chemotherapy reagents and prior therapies, were used to estimate HRs and to test for significance of the timing of events. The proportional hazard assumptions of the data were tested before Cox regression analysis. The method and results of the proportional hazard assumption test were shown in the supplementary information. Responses, survival rates, ORs and AEs were analyzed using Fisher's exact tests. Zelen's test for homogeneity of ORs across strata was performed to validate data. All reported P-values are two-sided, and CIs are at the 95% level.

### Role of the Funding Source

This trial was funded by New Taihe Biopharmaceutical Co., Ltd., Guangzhou, China and was designed and monitored by the Clinical Pharmacology Department of West China Center of Medical Sciences, Sichuan University. Data were gathered, analyzed, and interpreted by New Taihe as well as all authors and investigators. The corresponding author had full access to the study data and took full responsibility for the final decision to submit the report for publication.

## Author Contributions

Y. Z., M. L. and F. F. designed the study and coordinated the distributions of patients and the participant nurses in six hospitals. X. M., K. Z., Y. G., L. S., X. Z., W. Z. and X. X. collected data, performed the statistical analysis and prepared all tables and figures. X. M. and Y. S. wrote the main manuscript text. Y. S., H. J., J. G., Y. D., E. L., X. T. participated in the assignments of patients and follow up. All authors reviewed and approved the final manuscript.

## Supplementary Material

Supplementary Information Supplementary Information

## Figures and Tables

**Table 1:** Baseline Patient Demographic and Clinical Characteristics

	Chemotherapy plus rmhTNF (N = 265) No. (%)	Chemotherapy (N = 264) No. (%)
Age (years)		
Median	54.2	55.6
Range	27-69	28-70
Sex		
Male	170 (64)	164 (62)
Female	95 (36)	100 (38)
Smoking status		
Current and Former	140 (53)	143 (54)
Never	125 (47)	121 (46)
Stage of disease		
IIIB	175 (66)	174 (66)
IV	90 (34)	90 (34)
Histological type		
Adenocarcinoma	125 (47)	126 (48)
Squamous cell carcinoma Large cell carcinoma Others	140 (53)	138 (52)
ECOG PS		
0	69 (26)	74 (28)
1	196 (74)	190 (72)
Chemotherapy regimen		
Docetaxel + Carboplatin	241 (91)	246 (93)
Docetaxel + Cisplatin	24 (9)	18 (7)
Time since diagnosis (weeks)		
Median (Range)	10.2 (1–79)	11 (1–146)
Prior therapy		
Chemotherapy	148 (56)	145 (55)
Surgery	138 (52)	156 (59)
Biologic therapy (monoclonal antibodies, vaccines)	64 (24)	66 (25)
Radiotherapy	29 (11)	24 (9)
Follow-up anticancer therapy	N = 260	N = 260
Any therapy	150 (58)	181 (70)
Chemotherapy	116 (45)	130 (50)
Radiotherapy	15 (6)	21 (8)
Biologic therapy	44 (17)	59 (23)
Surgery	8 (3)	5 (2)
Unknown	27 (10)	8 (3)

ECOG PS: Eastern Cooperative Oncology Group Performance Status.

**Table 2:** Efficacy Results in Intent to Treatment Population (ITT)

	Chemotherapy plus rmhTNF N = 265 No. (%)	Chemotherapy N = 264 No. (%)	Odds Ratio (95% CI)	p value
Survival rate				
1 year	147 (55.5)	111 (42)	1.717 (1.217-2.422)	0.002
2 years	55 (20.8)	53 (20.1)	1.043 (0.683-1.591)	0.846
3 years	37 (14)	28 (10.6)	1.368 (0.81-2.309)	0.24
4 years	18 (6.8)	1 (0.4)	19.166 (2.54-144.645)	<0.001
Response				
RR (CR+PR)	102 (38.5)	73 (27.7)	1.637 (1.135-2.361)	0.008
CR	4 (1.5)	0	/	0.045
PR	98 (37)	73 (27.7)	1.535 (1.063-2.217)	0.022
SD	105 (39.6)	117 (44.3)	0.825 (0.583-1.165)	0.274
PD	58 (21.9)	74 (28)	0.719 (0.484-1.069)	0.103
Response duration (Month)				
First quartile	3.6	2.8	/	<0.001
Median	5.8	4.1	/	<0.001
Third quartile	11	9.8	/	<0.001

RR: Response Rate, CR: Complete Response, PR: Partial Response, SD: Stable Disease, PD: Progressive Disease, RR = CR+PR, CI: Confidence Intervals.

**Table 3:** Summary of Reported Adverse Events (AEs) by Grade

	Chemotherapy plus rmhTNF (n = 261)			Chemotherapy (n = 259)		
	All Grade No. (%)	Grade 3 No. (%)	Grade 4 No. (%)	All Grade No. (%)	Grade 3 No. (%)	Grade 4 No. (%)
AEs Experienced by >10% of Patients						
Diarrhea	256 (97)	1 (<1)	0	259 (98)	3 (1.1)	0
Rash	256 (97)	1 (<1)	2 (<1)	264 (100)	1 (<1)	0
Albuminuria	255 (96)	1 (<1)	0	262 (99)	2 (<1)	0
Arrhythmia	254 (96)	0	0	262 (99)	0	0
Infection (Non-neutropenia)	246 (93)	2 (<1)	0	262 (99)	1 (<1)	0
ALT Increase	246 (93)	5 (2)	0	248 (94)	8 (3)	0
AST Increase	234 (89)	3 (1)	1 (<1)	247 (94)	2 (<1)	1 (<1)
Stomatitis	246 (93)	0	0	264 (100)	1 (<1)	0
Thrombocytopenia	207 (78)	8 (3)	5 (2)	182 (69)	13 (5)	11 (4)
Fatigue	185 (70)	5 (2)	0	169 (64)	4 (1)	0
Myalgia	185 (70)	1 (<1)	0	259 (98)	0	0
Flu-like symptoms	170 (64)	10 (4)	1 (<1)	264 (100)	0	0
Pyrexia	162 (61)	16 (6)	8 (3)	248 (94)	0	0
Neutropenia	133 (50)	34 (13)	8 (3)	121 (46)	21 (8)	16 (6)
Leukopenia	122 (46)	29 (11)	13 (5)	66 (25)	24 (9)	13 (5)
Nausea	117 (44)	8 (3)	0	63 (24)	24 (9)	3 (1)
Vomiting	115 (43)	5 (2)	0	58 (22)	15 (6)	0
Abdominal Pain	87 (33)	0	0	74 (28)	0	0
Chest Pain	85 (32)	0	0	58 (22)	0	0
AEs of Special Interest						
Myalgia	185 (70)	1 (<1)	0	259 (98)	0	0
Flu-like symptoms*	170 (64)	10 (4)	1 (<1)	264 (100)	0	0
Pyrexia*	162 (61)	16 (6)	8 (3)	248 (94)	0	0
Febrile Neutropenia	16 (6)	0	0	21 (8)	0	0
Hematemesis *	16 (6)	0	3 (1)	0	0	0
Hypotension	2 (<1)	2 (<1)	0	0	0	0
Pulmonary Embolus	1 (<1)	1 (<1)	0	1 (<1)	1 (<1)	0
Cardiac event	1 (<1)	1 (<1)	0	2 (<1)	2 (<1)	0

ALT: Alanine Transaminase, AST: Aspartate Aminotransferase, *p<0.05 Comparison of Grade 3 and 4.
