# Erratum: Recombinant Mutated Human TNF in Combination with Chemotherapy for Stage IIIB/IV Non-Small Cell Lung Cancer: A Randomized, Phase III Study

**DOI:** 10.1038/srep46953

**Published:** 2018-03-19

**Authors:** Xiaowen Ma, Yang Song, Kuo Zhang, Lei Shang, Yuan Gao, Wei Zhang, Xiaochang Xue, Huimin Jia, Jian Geng, Wei Zhou, Yazheng Dang, Enxiao Li, Xinyu Ti, Fulin Fan, Yingqi Zhang, Meng Li

Correction to: Scientific Reports 5: Article number: 991810.1038/srep09918; published online: 04 21 2015; updated: 03 19 2018

The original version of this Article contained a typographical error in the volume number ‘5’ was incorrectly given as ‘4’. This error has now been corrected in the PDF and HTML versions of the Article.

