# Supplementary material for: Recombinant Mutated Human TNF in Combination with Chemotherapy for Stage IIIB/IV Non-Small Cell Lung Cancer: A Randomized, Phase III Study
Source: Sci Rep. 2015 Apr 21;4:9918. doi: 10.1038/srep09918 (PMC4404801; doi:10.1038/srep09918)
Supplement: Supplementary Information [file srep09918-s1.pdf]

SUPPLEMENTARY INFORMATION

Recombinant Mutated Human TNF in Combination with Chemotherapy for Stage IIIB/IV Non-Small Cell Lung Cancer: A Randomized, Phase III Study

Xiaowen Ma[1*], Yang Song[2*], Kuo Zhang[1*], Lei Shang[3*], Yuan Gao[1], Wei Zhang[1], Xiaochang Xue[1], Huimin Jia[4], Jian Geng[5], Wei Zhou[6], Yazheng Dang[7], Enxiao Li[8], Xinyu Ti[9], Fulin Fan[10], Yingqi Zhang[1], Meng Li[11]

[1]State Key Laboratory of Cancer Biology, Biotechnology Center, School of Pharmacy, The Fourth Military Medical University, Xi'an, China, [2]Department of Oncology, Tangdu Hospital, The Fourth Military Medical University, Xi'an, China, [3]Department of Health Statistics, School of Public Health, The Fourth Military Medical University, Xi'an, China, [4]The Affiliated Cancer Hospital of Xinjiang Medical University, Urumqi, China, [5]Department of Medical Oncology, General Hospital of Nanjing Military Command, Medical School of Nanjing University, Nanjing, China, [6]General Hospital of Ningxia Medical University, Yinchuan, China, [7]Cancer Center, The 323 Hospital of People's Liberation Army, Xi’an, China, [8]Department of Medical Oncology, First Affiliated Hospital of Medical College, Xi’an Jiaotong University, Xi’an, China, [9]Department of Respiratory Medicine, Xijing Hospital, The Fourth Military Medical University, Xi'an, China, [10]New Taihe Biopharmaceutical Co., Ltd., Guangzhou, China.

[11]Department of Pharmacogenomics, School of Pharmacy, The Fourth Military Medical University, Xi'an, China,

* These authors contributed equally to this work.

Correspondence and requests for materials should be addressed to: Meng Li and Yingqi Zhang, The Fourth Military Medical University, 169 Changle West Rd, Xi'an, China, 710032. Tel/Fax: +86 29 84774773. E-mail: lemon781106@hotmail.com (M. L.), zhangyqh@fmmu.edu.cn (Y. Z.).

The proportional hazard assumption test

Method

The proportional hazard assumptions of the data were tested using the log-log survivor plot[1]. The log-log survivor plot is a plot defining *log (-log S (t))* as the vertical axis, and *log t* as the horizontal axis, where *t* denotes study time, *S (t)* denotes survival rate in the Kaplan-Meier analysis. In this test, the proportional hazard assumption has not been violated if the plotted lines for a given independent variable are reasonably parallel.

Results

According to the data in Kaplan-Meier analysis, the log-log survivor plots of overall

survival (Fig. S A) and progression free survival (Fig. S B) for all of 529 patients were plotted. The plotted lines in the study period are approximately parallel. Therefore, the proportional hazard assumption for our data set was valid and the proportional hazards model (Cox regression) could be used for analysis.

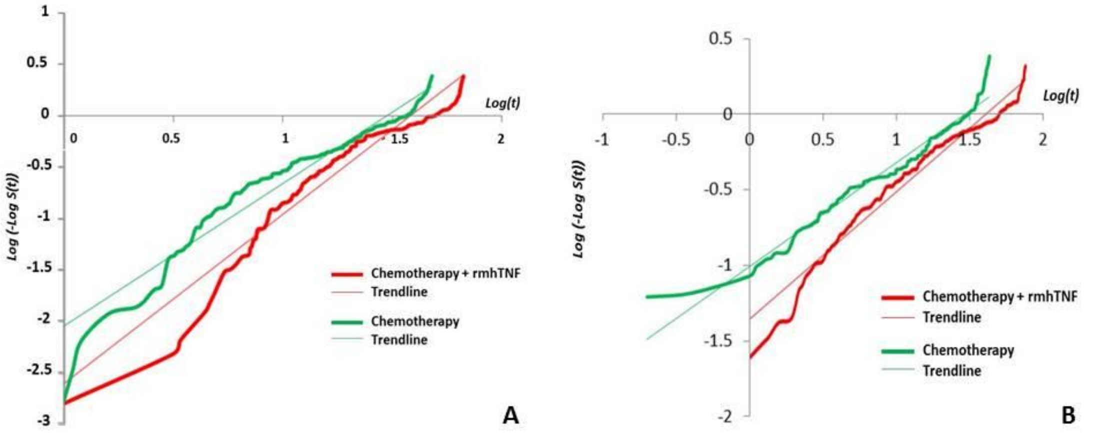

Figure S. The log-log survivor plots of the proportional hazard assumption test. t denotes study time, S (t) denotes survival rate in the Kaplan-Meier analysis. (A) The log-log survivor plot for the overall survival analysis. (B) The log-log survivor plot for the progression free survival analysis.

Reference

1. Shenyang Guo. Survival Analysis (ed. Tony Tripodi) Ch. 4, 82-82 (Oxford University Press, 2010).
